# Chemical Composition and Toxicity against *Sitophilus zeamai s* and *Tribolium castaneum* of the Essential Oil of *Murraya exotica* Aerial Parts

**DOI:** 10.3390/molecules15085831

**Published:** 2010-08-25

**Authors:** Wei Qing Li, Cai Hong Jiang, Sha Sha Chu, Ming Xue Zuo, Zhi Long Liu

**Affiliations:** 1 College of Life Sciences, Beijing Normal University, Haidian District, Beijing 100875, China; E-Mails: liweiqing0102@sina.com (W.Q.L); mxzuo@bnu.edu.cn (M.X.Z.); 2 College of Chemical and Biological Engineering, Changsha University of Science and Technology, Changsha 41004, China; 3 Department of Entomology, China Agricultural University, Haidian District, Beijing 100094, China; E-Mails: jiangcaihong1987@163.com (C.H.J.); chushasha3421@126.com (S.S.C.)

**Keywords:** *Murraya exotica*, *Sitophilus zeamais*, *Tribolium castaneum*, fumigant, contact toxicity, essential oil

## Abstract

In our screening program for new agrochemicals from Chinese medicinal herbs, *Murraya exotica* was found to possess insecticidal activity against the maize weevil, *Sitophilus zeamais* and red flour beetle, *Tribolium castaneum*. The essential oil of aerial parts of *M. exotica* was obtained by hydrodistillation and investigated by GC and GC-MS. The main components of *M. exotica* essential oil were spathulenol (17.7%), α-pinene (13.3%), caryophyllene oxide (8.6%), and α-caryophyllene (7.3%). Essential oil of *M. exotica* possessed fumigant toxicity against *S. zeamais* and *T. castaneum* adults with LC_50_ values of 8.29 and 6.84 mg/L, respectively. The essential oils also show contact toxicity against *S. zeamais* and *T. castaneum* adults with LD_50_ values of 11.41 and 20.94 μg/adult, respectively.

## 1. Introduction

*Sitophilus* and *Tribolium* species are two of the major pests of stored grains and grain products in the tropics and subtropics [1]. Control of stored product insects relies heavily on the use of synthetic insecticides and fumigants, which has led to problems such as environmental disturbances, increasing costs of application, pest resurgence, pest resistance to pesticides and lethal effects on non-target organisms, in addition to direct toxicity to users [2]. Fumigation plays a very important role in insect pest elimination in stored products [3]. Plant essential oils and their components have been shown to possess potential for development as new fumigants and they may have advantages over conventional fumigants in terms of low mammalian toxicity, rapid degradation and local availability [4]. Essential oils derived from more than 75 plant species have been evaluated for fumigant toxicity against stored product insects so far [5].

Botanical pesticides have the advantage of providing novel modes of action against insects that can reduce the risk of cross-resistance as well as offering new leads for design of target-specific molecules [2,6]. During our screening program for new agrochemicals from local wild plants and Chinese medicinal herbs, the essential oil from aerial parts of *Murraya exotica* L. have been found to possess insecticidal activity towards the maize weevil, *Sitophilus zeamais* (Motsch) and red flour beetle, *Tribolium castaneum* Herbst. 

*Murraya exotica* belongs to the family Rutaceae and is an evergreen shrub or occasionally a small tree, usually 2 to 3 m in height but reaching 8 m and 13 cm in stem diameter. Its flowers are few, white, and very fragrant [7]. It is commonly cultivated in gardens in many tropical and subtropical countries for its glossy green foliage and large clusters of fragrant flowers [7]. An infusion of the leaves and flowers of *M. exotica* is tonic and stomachic. It is said to be aromatic, refrigerant, digestive, and beneficial in rheumatic fever, coughs, giddiness, hysteria, thirst, and burning of the skin [8,9]. Phytochemical studies on *M. exotica* revealed the presence of flavonoids, coumarins, phytosterols, alkaloids and volatile oil [10,11,12,13,14,15,16,17,18,19,20,21,22,23,24,25]. The chemical composition of essential oil of *M. exotica* has been widely studied [26,27,28,29,30,31]. The acetone extract of *M. exotica* leaves showed antifeedant activity against the 3^rd^ larvae of *Spodptera litura* [32] while the ethanol extract of this plant was found to possess antifeedant activity against aphids (*Myzus persicae* and *Lipaphis erysimi*) [33]. Moreover, murraxocin (coumarin), derived from *M. exotica* showed insecticidal activity against three important forest insect pests (*Plecoptera reflexa*, *Clostera cupreata* and *Crypsiptya coclesalis*). However, no report on insecticidal activity of essential oil of *M. exotica* against stored product insects was available.

## 2. Results and Discussion

### 2.1. Chemical composition of the essential oil

A total of 36 components were identified in the essential oil of *M. exotica*, accounting for 99.5% of the total oil). The chemical composition of the essential oil is shown in Table 1. The main constituents of the oil were spathulenol (17.7%), α-pinene (13.3%), caryophyllene oxide (8.6%), α-caryophyllene (7.3%), and bicyclogermacrene (7.1%), followed by 1,2,3,5,6,7,8,8a-octahydro-1-methyl-6-methylene-4-(1-methylethyl)-naphthalene (6.5%) and γ-selinene (5.3%). 

**Table 1:** Chemical composition of the essential oil of *Murraya exotica .*

Compounds	RI *	Relative content (%)
α-Pinene	931	13.2
α-Phellandrene	1005	0.2
δ-3-Carene	1010	0.5
ρ-Cymene	1024	1.1
Linalool	1094	0.5
α-Terpineol	1191	0.7
Thymol methyl ether	1225	0.2
p-Menth-1(7)-en-2-one	1238	0.3
Phellandral	1281	0.8
Bornyl acetate	1285	1.8
Thymol	1292	2.2
4-Vinylguaiacol	1311	0.3
Eugenol	1356	1.2
α-Copaene	1374	1.7
β-Patchoulene	1388	1.0
β-Elemene	1391	0.9
1,2,3,5,6,7,8,8a-octahydro-1-methyl-6-methylene-4-(1-methylethyl)-Naphthalene	1401	6.5
Caryophyllene	1420	4.5
Calarene	1432	0.7
α-Caryophyllene	1454	7.3
γ-Selinene	1455	5.3
*allo*-Aromadendrene	1458	0.2
γ-Muurolene	1473	0.7
β-Selinene	1475	1.5
α-Amorphene	1479	0.5
Germacrene D	1481	0.8
Bicyclogermacrene	1499	7.1
1ξ,6ξ,7ξ-Cadina-4,9-diene	1502	0.9
δ-Cadinene	1523	4.4
Cadina-1,4-diene	1533	0.5
Eudesma-3,7(11)-diene	1536	0.2
α-Calacorene	1543	0.7
*cis*-Nerolidol	1535	1.2
Spathulenol	1578	17.7
Caryophyllene oxide	1583	8.6
α-Cadinol	1640	3.6
Total		99.5

* RI, retention index as determined on a HP-5MS column using the homologous series of *n*-hydrocarbons.

**Table 2:** Insecticidal activity of the essential oil of *Murraya exotica* against *Sitophilus zeamais* (SZ) and *Tribolium castaneum* (TC) adults.

Insects	Essential oil	Contact toxicity (7 d)		Fumigant toxicity (7 d)	
		LD_50_ (μg/adult)	95% confidence limits	LC_50_ (mg/L)	95% confidence limits
	*M. paniculata*	11.41	10.77-12.12	8.29	7.49-9.23
SZ	Pyrethrum extract	4.29	3.86-4.72	-	-
	MeBr	-	-	0.67*	-
	*M. paniculata*	20.94	19.62-22.53	6.84	6.15-7.52
TC	Pyrethrum extract	0.36	0.32-0.41	-	-
	MeBr	-	-	1.75*	-

* data from Liu and Ho [1].

The results were quite different from the previous reports. These differences might have been due to harvest time and local, climatic and seasonal factors, as well as duration of the storage of the medicinal herbs. For example, the major constituents of volatile oil collected from Guangxi Autonomous District were bicyclogemacrene (26.0%), β-caryophyllene (20.8%), α-caryophyllene (5.8%), δ-cadinene (4.7%), and spathulenol (4.3%) [26]. Rina *et al.* [28] determined the chemical composition of essential oil of *M. exotica* leaves and flowers collected from India. The results indicated that the leaf oil showed (*E*)-nerolidol (27.8%), α-zingiberene (10.0%), β-caryophyllene (9.7%), (*E*, *E*)-farnesol (8.9%) and δ-elemene (5.1%) as the major constituents, while the flower oil showed (*E*,*E*,*E*)-α-springene (23.8%), (*E*)-nerolidol (18.7%), (*E*,*E*)-α-farnesene (13.2%), methyl palmitate (6.8%) and germacrene B (5.9%) as the major constituents. However, the essential oil of *M. exotica* harvested from Cuba possessed β-caryophyllene (24.1%) as major constituent [29] while Olawore *et al.* [30] found that the principal constituents of the leaf essential oil of *M. exotica* grown in Nigeria were β-cyclocitral (22.9%), methyl salicylate (22.4%), and *trans*-nerolidol (11.7%), while the most abundant constituent of the fruit essential oil was β-caryophyllene (43.4%) followed by (-)-zingiberene (18.9%) and germacrene D (8.3%). However, the essential oils of fresh flowers, leaves and fruits of *M. exotica* cultivated in Egypt possessed a major component, α-pinene (88.9%, 62.5%, and 80.4%, respectively [31]. Further studies on plant cultivation and essential oil standardization are needed because chemical composition of the essential oil varies greatly with the plant population.

### 2.2. Insecticidal activity

The essential oil of *M. exotica* showed contact toxicity against *S. zeamais* and *T. castaneum* adults with LD_50_ values of 11.41 and 20.94 μg/adult, respectively (Table 2). Compared with the famous botanical insecticide, pyrethrum extract, the essential oil was 2.5 times less active against the maize weevils and 58 times less active against the red flour beetles because pyrethrum extract displayed LD_50_ value of 4.29 and 0.36 μg/adult, respectively (Table 2). The essential oil also possessed strong fumigant activity against *S. zeamais* and *T. castaneum* adults with LC_50_ values of 8.29 and 6.84 mg/L, respectively (Table 2). The currently used grain fumigant, methyl bromide (MeBr) was reported to have fumigant activity (24 h) against *S. zeamais* and *T. castaneum* adults with LC_50_ values of 0.67 and 1.75 mg/L, respectively [33]. The essential oil was 12 times less toxic to the maize weevils and 4 times less toxic to the red flour beetles compared with the commercial fumigant MeBr. However, considering the currently used fumigants are synthetic insecticides, fumigant activity of the essential oil of *M. exotica* is quite promising and it shows potential to be developed as a possible natural fumigant for control of stored product insects. 

In traditional Chinese medicine, *M. exotica* is used to treat many problems such as rheumatic fever, coughs, giddiness, hysteria and skin diseases [8]. It seems that this medicinal herb is quite safe to human consumption. However, no experimental data about the safety of this herb is available so far. In the previous studies, one of the main components of the essential oil (α-pinene) has been demonstrated contact and fumigant toxicity against several species of insects (cockroaches, head lice, cotton rootworm and stored product beetles and weevils) and mites [36,37,38,39,40]. However, no data on insecticidal activities of the other main components (spathulenol, caryophyllene oxide, α-caryophyllene, bicyclogermacrene) of the essential oil was available. The isolation and identification of the bioactive compounds in the essential oil of *M. exotica* aerial parts are of utmost importance so that their potential application in controlling stored-product pests can be fully exploited.

## 3. Experimental

### 3.1. Plant material

Dried aerial parts (15 kg of leaves, young floriferous stems) of *M. exotica* were purchased from Puning Chinese Medicinal herbs Market (Guangdong 515300, China). The aerial parts were ground to a powder. The species was identified, and the voucher specimens (BNU-liuzhilong-2009-08-29-028) were deposited at the Herbarium (BNU) of College of Life Sciences, Beijing Normal University.

### 3.2. Insects

The maize weevil, *S . zeamais* and red flour beetle, *T. castaneum* were obtained from laboratory cultures maintained in the dark in incubators at 29-30 ºC and 70-80% relative humidity. The red flour beetle, *T . castaneum* was reared on wheat flour mixed with yeast (10:1, w/w) while *S. zeamais* was reared on whole wheat at 12-13% moisture content. Unsexed adult weevils/beetles used in all the experiments were about 2 weeks old. 

### 3.3. Essential oil distillation

The ground powder of *M. exotica* was subjected to hydrodistillation using a modified Clevenger-type apparatus for 6 h and extracted with *n*-hexane. Anhydrous sodium sulphate was used to remove water after extraction. Essential oil was stored in airtight containers in a refrigerator at 4 ºC. The oil yield of *M . paniculata* was 0.15% v/w.

### 3.4. Gas chromatography and mass spectrometry

Gas chromatographic analysis was performed on an Agilent 6890N instrument equipped with a flame ionization detector and HP-5MS (30 m × 0.25 mm × 0.25 μm) capillary column, while the essential oil components were identified on an Agilent Technologies 5973N mass spectrometer. The GC settings were as follows: the initial oven temperature was held at 60 ºC for 1 min and ramped at 10 ºC min^−1^ to 180 ºC for 1 min, and then ramped at 20 ºC min^−1^ to 280 ºC for 15 min. The injector temperature was maintained at 270 ºC. The samples (1 μL) were injected neat, with a split ratio of 1:10. The carrier gas was helium at flow rate of 1.0 mL min^−^^1^. Spectra were scanned from 20 to 550 m/z at 2 scans s^-1^. Most constituents were identified by gas chromatography by comparison of their retention indices with those of the literature or with those of authentic compounds available in our laboratories. The retention indices were determined in relation to a homologous series of *n*-alkanes (C_8_–C_24_) under the same operating conditions. Further identification was made by comparison of their mass spectra on both columns with those stored in NIST 05 and Wiley 275 libraries or with mass spectra from literature [34]. Component relative percentages were calculated based on GC peak areas without using correction factors. 

### 3.5. Fumigant toxicity

The fumigant activity of the essential oil against *S. zeamais* and *T. castaneum* adults was tested as described by Liu and Ho [1]. A serial dilution of the essential oil (six concentrations) was prepared in *n*-hexane. A Whatman filter paper (diameter 2.0 cm) were each impregnated with 20 μL dilution, and then placed on the underside of the screw cap of a glass vial (diameter 2.5 cm, height 5.5 cm, volume 25 mL). The solvent was allowed to evaporate for 30 s before the cap was placed tightly on the glass vial, each of which contained 10 insects inside to form a sealed chamber. Preliminary experiments demonstrated that 30 s was sufficient for the evaporation of solvents. *n*-Hexane was used as a control. Five replicates were carried out for all treatments and controls, and they were incubated for 24 h. The insects were then transferred to clean vials with some culture media and returned to the incubator and observed daily for determination of end-point mortality, which was reached after one week. The experiments were repeated in three times. The LC_50_ values were calculated by using Probit analysis [35]. 

### 3.6. Contact toxicity

The contact toxicity of the essential oil against *S. zeamais* and *T. castaneum* adults was measured as described by Liu and Ho [1]. A serial dilution of the essential oil (five concentrations) was prepared in *n*-hexane. Aliquots of 0.5 μL of the dilutions were applied topically to the dorsal thorax of the insects. Controls were determined using *n*-hexane. Both treated and control insects were then transferred to glass vials (10 insects/vial) with culture media and kept in incubators. Mortality of insects was observed daily until end-point mortality was reached one week after treatment. The experiments were repeated in three times. The LD_50_ values were calculated by using Probit analysis [35]. Pyrethrum extract (25% pyrethrine I and pyrethrine II) was purchased from Fluka Chemika GmbH and used as a positive control.

## 4. Conclusions

Based on mass screening, essential oil of *M. exotica* was examined for their insecticidal activity against the two grain storage insects. The essential oil possessed strong fumigant toxicity against adults of the two grain storage insects, although it was 10 times and 4 times less toxic to the maize weevil and red flour beetle, respectively compared to commercial fumigant MeBr. The essential oil also showed contact toxicity against adults of the two grain storage insects. These findings, considered together, suggest that the essential oil shows potential for development as a natural fumigant for stored products.
